# Alkali Activation of Waste Clay Bricks: Influence of The Silica Modulus, SiO_2_/Na_2_O, H_2_O/Na_2_O Molar Ratio, and Liquid/Solid Ratio

**DOI:** 10.3390/ma13020383

**Published:** 2020-01-14

**Authors:** R. A. Gado, Marek Hebda, Michal Łach, Janusz Mikuła

**Affiliations:** 1 Department of Refractories, Ceramic and Building Materials, National Research Center (NRC), 12311 Dokki Cairo, Egypt; 2 Institute of Materials Engineering, Faculty of Materials Engineering and Physics, Cracow University of Technology, 31-155 Kraków, Poland; mhebda@pk.edu.pl (M.H.); mlach@pk.edu.pl (M.Ł.); jamikula@pk.edu.pl (J.M.)

**Keywords:** geopolymer, waste fired clay bricks (Grog), silica modulus (Ms), H_2_O/Na_2_O molar ratio, liquid/solid ratio, geopolymerization rate

## Abstract

This study was conducted to investigate the influence of various reaction conditions, namely the silica modulus SiO_2_/Na_2_O, H_2_O/Na_2_O molar ratio, and liquid/solid ratio on the geopolymerization reaction of the waste fired clay bricks (Grog). The starting raw material and the generated geopolymer specimens produced by different geopolymerization reaction conditions have been characterized using different techniques: X-ray diffraction (XRD), Fourier-transform infrared spectroscopy (FTIR), scanning electron microscope (SEM) and thermal analysis. Furthermore, physico–mechanical characterization has been carried out through the determination of bulk density, porosity, water absorption, and compressive strength for each sample at interval curing times of up to 28 days. The results indicated that the geopolymerization system of the waste fired clay bricks is influenced by the investigated reaction conditions at room temperature. The compressive strength of the geopolymer sample produced at optimum conditions increased significantly by up to 37.5 MPa, in comparison with 4.5 MPa for other conditions. Finally, an optimum recommendation and useful conclusions concerning the recycling and utilization of this waste material through the geopolymerization process are made for compatibility with construction applications.

## 1. Introduction

Waste fired clay bricks (Grog) are classified as a waste material generated by the clay bricks manufacture process and are accumulated in large amounts. Annual production of this industrial by-product makes up approximately 5 wt.% of the total mass production of clay bricks. Hence, this waste material causes many critical environmental problems due to its disposal over a large landfill area as well as contamination of the surrounding region. In addition, Grog is classified chemically as an alumina-silicate material that is related to its original formation as a result of the mixing process of sand and raw clay (a mixture of different clay minerals) during the production of clay bricks in a factory [1,2].

Geopolymer materials are considered to be a class of inorganic polymer materials consisting mainly of an amorphous phase composed of a silicon or aluminum atom linked to four oxygen atoms by covalent bonds [3]. A three-dimensional network microstructure product was generated upon alkaline activation of an aluminosilicate source material at an ambient or higher temperature through an alkaline activation reaction called geopolymerization. An alkaline media solution is most often used with a mixture of liquid sodium silicate (Na_2_SiO_3_) and sodium hydroxide (NaOH) at different molar ratios. Many previous studies [4,5,6,7,8] showed that a geopolymer matrix can be produced using geopolymerization involving many different raw silico-aluminates and alumino-silicates materials, as well as industrial by-product or waste materials essentially composed of alumina silicate source materials. Generally, geopolymers are attractive materials for this process due to their superior physico–mechanical properties and ability to achieve high durability, thermal stability, and acid resistance in comparison with traditional and conventional cementitious based materials. Schmucker and MacKenzie [9] showed that the composition of geopolymers is practically unchanged during firing to a high temperature of 1200 °C. Furthermore, due to a notably lower calcium content, a geopolymer matrix is much more acid resistant than a cement-based binder material [10].

The basic geopolymerization process generally involves three reaction steps: the dissolution of the starting alumina silicate material at high pH; dissolved species hydrolysis; and condensation between tetrahedral (AlO_4_) and (SiO_4_) units of the gel phase, which is typically the “X-ray amorphous phase”. Furthermore, alkali hydroxide is required for the dissolution step of alumino-silicates material while a sodium silicate solution acts as a binder agent, alkali activator, and a dispersant or a plasticizer medium. The properties of geopolymer products are significantly affected by the specific surface area and chemical composition of the initial raw materials and the relative amount of alkali activator solution used during the initial period of the geopolymerization process [11]. Moreover, the liquid-to-solid (L/S) ratio and the ratio of Na_2_SiO_3_-to-NaOH have a significant effect on the compressive strength of the final geopolymer product, as well as influencing the workability of the geopolymer paste or slurry [12]. Meanwhile, some studies have been carried out to establish the effect of water content on the geopolymerization reaction using different base materials. The water content has been reported to play an active part in enhancing the dissolution process of Al-Si species, not by taking part in the polymerization reaction but by being released during the polymerization reaction of the alkali-activated product. The geopolymerization process rate is influenced by different reaction parameters such as alkali concentration, water content, curing temperature, liquid/solid ratio, Si/Al ratio, pH, and the type of activators used, which are also important factors in developing the physico–mechanical properties of any geopolymer product. Furthermore, the compressive strength of any geopolymer matrix is a common indicator of the success of any geopolymerization process [13].

Generally, fresh geopolymer paste prepared with a high liquid/solid ratio has a low viscosity system, while geopolymer paste with a low liquid/solid ratio results in a high viscosity system and consequently, also affects its workability [11]. Moreover, the variation in the liquid/solid ratio of any geopolymer system will have a direct influence on the dissolution rate of the starting material, which significantly accelerates or retards the formation of binder and the geopolymerization rate [14]. On the other hand, different ratios of sodium silicate to sodium hydroxide mainly affect the pH conditions of the prepared alkaline solution and thus could have some effects on the properties of the resulting geopolymer product. In addition, it is well-known that variation of the silica modulus Ms = SiO_2_/Na_2_O molar ratio significantly modifies the degree of polymerization of the dissolved species in a silicate solution, which plays a significant part in determining the structure and properties of geopolymers [15].

Grog is an alumino-silicate by-product waste material, and its recycling has significant impacts on the environmental and industrial sectors. In general, there is a variation in the chemical composition of grog for each region based on the raw material mineralogy used, but the majority of the composition is still an alumino-silicate by-product. Recycling this material for the production of geopolymer products is worthy of environmental consideration [16]. In the previous study, Grog-based geopolymers have been produced and synthesized with the different inclusion ratio of another industrial by-product known as granulated blast-furnace slag, which is classified as a high alkali-activation material [2]. Therefore, the aim of this study was to investigate the influence of key parameters on the geopolymerization reaction of the Grog itself without the mixing or inclusion of other alkali-activation materials. In this analysis, the parameters are the silica modulus SiO_2_/Na_2_O, H_2_O/Na_2_O molar ratio, and liquid/solid ratio. Moreover, the chemical and microstructural properties of synthesized geopolymer specimens were investigated with respect to their physico–mechanical properties, phase composition, and microstructure. The results obtained from this study will contribute to the development and improvement of optimum geopolymerization condition parameters for this type of waste material. Moreover, the aim was to provide a potential replacement of traditional cementitious binders by using an alternative binder material based on a safe recycling process, which has practical implications and advantages in the environmental sector.

## 2. Experimental and Methodology

### 2.1. Starting Materials

The starting material used for the geopolymer synthesis process was an industrial by-product which is well known as Grog. The material was provided by an Egyptian clay bricks manufacture (Misr-Brick Company, Helwan, Giza, Egypt). The Grog was ground in a ball mill (Fritsch planetary-Pulverisette 6, FRITSCH GmbH, Idar-Oberstein, Germany) for one hour to obtain a powder with small particle sizes. X-ray fluorescence (XRF, Malvern Panalytical B.V., Almelo, Netherlands) was used to determine the chemical oxide compositions of the starting material. In addition, the crystalline mineral phases were identified by X-ray diffraction technique using a Panalytical X’pert Pro (Malvern Panalytical Ltd., Malvern, UK) diffractometer with a Ni filter, as well as Cu Kα radiation with a wavelength of 1.5406 Å, at a scan speed of 0.5 min^−1^, operated at 40 kV, 40 mA, and a 2 theta range of 4–70°. In addition, the infrared spectrum (FTIR) was recorded in the wavelength range of 400–4000 cm^−1^ by using a JASCO FT/IR-6100 (FRITSCH GmbH, Idar-Oberstein, Germany). The particle size distribution was determined by the laser diffraction wet-dispersion method, in accordance with ISO 13320, using Fritsch Analysette 22 Micro-Tec plus (Fritsch GmbH, Idar-Oberstein, Germany). Blain surface area was measured by an equipment supplied from ELE International (Leighton Buzzard, UK). Scanning electron microscopy (SEM, JEOL-JSM-5510LV, JEOL Ltd., Tokyo, Japan) coupled with energy dispersive spectroscopy (EDS) model IXRF 500 (NTC Tech Inc., Rancho Cordova, CA, USA), was used for investigation of the morphology structure of the Grog. The investigated samples were coated with a thin gold layer using sputter coater to make them conductive and ensure there was no arching or image instability during the SEM observation process [17].

The dissolution rate of Si and Al from the starting aluminosilicate source material plays a crucial role in geopolymerization reactions. Hence, the extent of dissolution rate of the source materials in alkaline mediums at low solid/solution ratios can be used for prediction of their behavior at high solid/solution ratios. Therefore, the reactivity of the starting material (Grog) for geopolymerization was assessed by testing the dissolution extents of Si and Al species from the solid source materials in sodium hydroxide, through inductively coupled plasma atomic emission spectroscopy (ICP-OES). The leaching of the Grog was conducted by immersing 1.00 (±0.0001) g of the dry powder sample in 40 mL of 1 M NaOH solution in a glass beaker and magnetically stirring at room temperature. After 5 h of mechanical stirring, the solution was acidified, filtered, and diluted. Afterwards, ICP-OES was employed to analyze the Al and Si concentrations in the filtrated solution [18]. Commercially available sodium hydroxide (NaOH flakes, PCC Rokita, Brzeg Dolny, Poland), distilled water, and liquid sodium silicate with a specific gravity of 1.46 g/cm^3^ at 20 °C and an initial silica modulus ratio (Ms = SiO_2_/Na_2_O) of 2.50 were used for the preparation of the different alkaline activator solutions and were allowed to cool at room temperature (25 ± 2 °C) for 24 h prior to the geopolymer preparation process.

### 2.2. Preparation of Alkaline Activator Solutions

Different alkaline activator solutions were prepared by mixing different proportions of sodium hydroxide solution (NaOH, 10 M) and sodium silicate solution (Ms = 2.5). To investigate the effects of the silica modulus molar ratio, the total silica modulus ratios of the prepared alkaline activator solutions were 0.50, 0.75, 1.00, 1.25, 1.50, 2.00, and 2.50 with a fixed H_2_O/Na_2_O molar ratio [19]. Moreover, the influence of different H_2_O/Na_2_O molar ratios on the geopolymerization reaction of the waste fired clay bricks was investigated with the optimum silica modulus molar ratio (Ms = SiO_2_/Na_2_O) obtained in terms of the highest value of mechanical performance. The H_2_O/Na_2_O molar ratio ranged from 10–20, and the proportion of solid powder to alkaline activator solution by mass was fixed at a liquid/solid ratio of 0.35 to allow a full geopolymerization process to occur [12]. In addition, an investigation of the influence of the liquid to solid ratio on the geopolymerization reaction of Grog at the optimum silica modulus (Ms = SiO_2_/Na_2_O) and H_2_O/Na_2_O molar ratio was carried out. A total of sixteen different geopolymer mixes were formulated in the current study. Moreover, the effects of a different total silica modulus (Ms = SiO_2_/Na_2_O), H_2_O/Na_2_O molar, and liquid to solid ratio (L/S) on the Grog geopolymerization reaction were monitored through determination of the setting time (initial and final setting time) as well as the characterization of generated geopolymer specimens to establish a recommendation for optimum geopolymerization reaction conditions.

### 2.3. Geopolymer Synthesis and Characterization Procedures

The synthesis of different geopolymer pastes was carried out using mixtures of starting source material (Grog) and different alkaline activator solutions. Immediately after adding the alkaline activator solution to the dry powder and mixing manually for about 5 min, the pastes were casted into 12.5 × 12.5 × 12.5 mm molds in two layers [20]. The molding process was followed by compacting vibrating, using a vibration table for approximately 2 min, and subsequent wrapping in plastic film sheets to avoid moisture evaporation at ambient temperature (25 °C). The molds were opened after 24 h and the de-molded specimens were preserved at ambient temperature until we tested their physico–mechanical properties at curing time intervals.

The method described by the American Society for Testing and Materials [21], based on Archimedes’ principle, was used to measure the bulk density and apparent porosity of the final geopolymer specimens [22]. The water absorption of all geopolymer specimens was calculated based on the percentage increase in the weight of the complete surface dry samples at a temperature of 80 °C in the oven for 24 h, as well as after the sample had been boiled for 2 h and submerged in water for 24 h [23]. Compressive strength tests were carried out using a MATEST (Model C-104 with Cyber-plus evolution program, MATEST S.p.A., Arcore, Italy) universal crushing machine, with a 0.05 KN/s loading rate. The average failure load per cross-sectional area (MPa) of the three geopolymer specimens were then recorded for the compressive strength values [20]. After that, the fractions of the geopolymer specimens were characterized by using various techniques: X-ray diffraction (XRD, Panalytical X’pert Pro, Malvern Panalytical Ltd., Malvern, UK); Fourier transforms infrared spectroscopy (FTIR, JASCO FT/IR-6100, FRITSCH GmbH, Idar-Oberstein, Germany); Scanning electron microscope (SEM, JEOL-JSM-5510LV, JEOL Ltd., Tokyo, Japan); Thermal analysis involving thermogravimetry and differential scanning calorimetry (TG & DSC, STA 409 CD (Netzsch) advance coupling techniques, NETZSCH-Gerätebau GmbH, Selb, Germany) as well as dilatometry curve of the geopolymer specimen produced by the optimum conditions. The calorimetric curves were recorded with a DSC using a closed alumina crucible (85 µL, with a 50 µm hole in the lid), with about 150 mg of a sample under a dynamic air atmosphere (80 mL/min). The temperature was in the range of 30–1000 °C at a heating rate of 10 °C/min. An empty closed alumina crucible was used as a reference. The apparatus was calibrated in accordance with the methodology presented in previous studies [24,25]. A dimensional change behavior test of the optimum geopolymer specimen was performed in a horizontal NETZSCH 402 C dilatometer. The process was carried out under the same conditions as the TG/DSC measurements. Moreover, the data were analyzed using Proteus software (ver. 5.2.0) supplied by Netzsch.

### 2.4. Monitoring the Geopolymerization Rate

The alkali activator solution is known to play a major role in the kinetics of the geopolymerization reaction [26]. The reaction rates of geopolymerization and hardening of synthesized geopolymer paste with a different silica modulus ratio, H_2_O/Na_2_O molar ratio, and L/S ratio were monitored by the direct determination of initial and final setting time using Vicat′s apparatus according to the ASTM methods [27] but during the test, different alkaline activator solutions were used instead of water. The initial and final setting times were estimated by measuring the penetration depth of the Vicat′s needles into geopolymer pastes.

## 3. Results and Discussions

### 3.1. Starting Material Characterization

**Figure 1:**
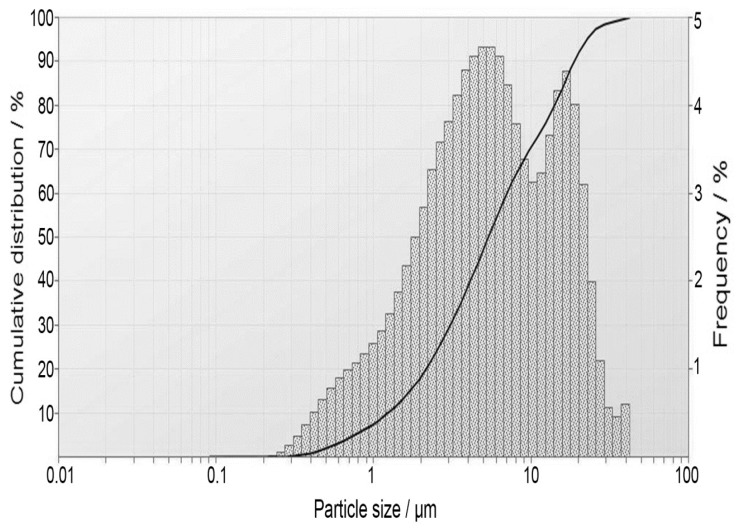
The particle size distribution of the starting material (Grog).

The main oxides composition of the starting raw material (Grog) is given in Table 1. The waste fired clay bricks composed mainly of aluminosilicate with some chemical oxides (Fe_2_O_3_, CaO, and Na_2_O). The measured specific gravity and Blaine surface area of the starting material were 2.38 g/cm^3^ and 7600 cm^2^/g, respectively. Moreover, the particle size distribution is presented in Figure 1 and the majority of the particle size distribution was recorded in the range of 0.5–10 μm.

**Figure 2:**
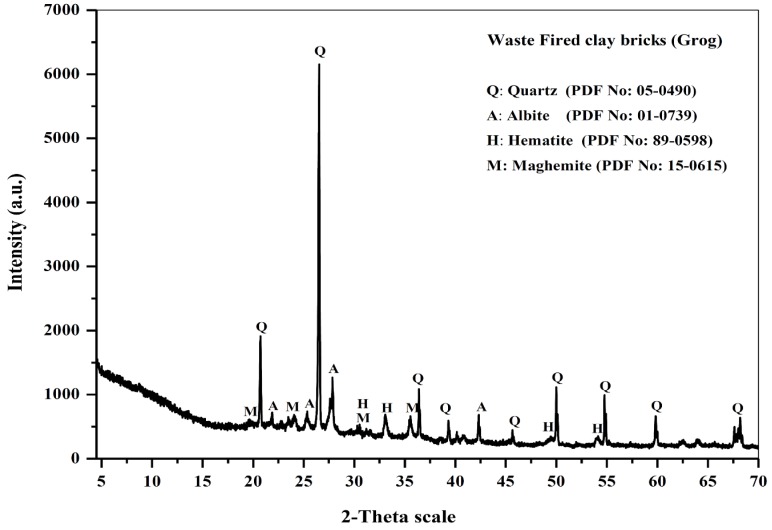
XRD pattern of the starting material (Grog).

The (XRD) pattern of the starting material, as shown in Figure 2, indicates that the major crystalline phase present was a quartz phase (SiO_2_), which was mainly related to sand addition during the raw meal preparation step for producing the clay brick units. In addition, the presence of some minor peaks corresponding to some minor constituents such as albite (NaAlSi_3_O_8_) and iron oxide in the form of hematite and maghemite minerals was observed. However, iron-bearing materials such as iron-rich geological material (40% Fe_2_O_3_), fly ash (10% Fe_2_O_3_), and ground granulated blast-furnace slag (0.5% Fe_2_O_3_) were used to produce geopolymers, as the presence of iron oxides might play important roles in the microstructure and overall properties of the product, although substitutions of Al by Fe have not yet been fully studied [28]. Otherwise, the XRD pattern does not show any crystalline phases related to the clay mineral structure, which completely transfers to an amorphous aluminosilicate structure generated upon the firing step at about 900 °C during clay brick manufacturing [29].

**Figure 3:**
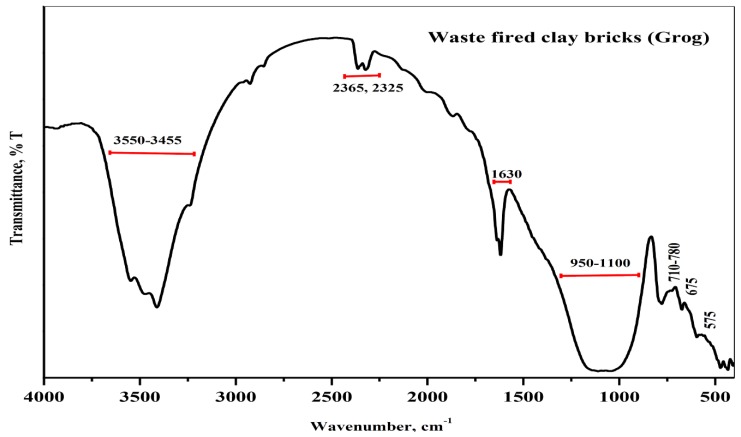
FTIR spectra of the starting material (Grog).

Figure 3 shows the FTIR spectra of the starting material. The main broad band centered at a wavenumber of around ≈ 1100 cm^−1^ and other bands located at 460 cm^−1^ were associated with asymmetric Si–O stretching in the tetrahedral structure [10]. Otherwise the bands at wavenumber 450–750 cm^−1^ related to the vibration mode of Al–O–Si bonds of any aluminosilicate powder material [30]. Moreover, other main broad bands at 3548 and 3455 cm^−1^ related to the adsorbed hydroxyl group associated with the band at 1630 cm^−1^ indicate the presence of a water molecule due to the humidity in the starting material. Furthermore, the presence of two minor bands approximately at wavenumbers 2925 and 2856 cm^−1^ corresponded to the antisymmetric stretching vibration and symmetric stretching vibration of the methylene group (CH_2_) arising from the presence of organic impurities [2].

**Figure 4:**
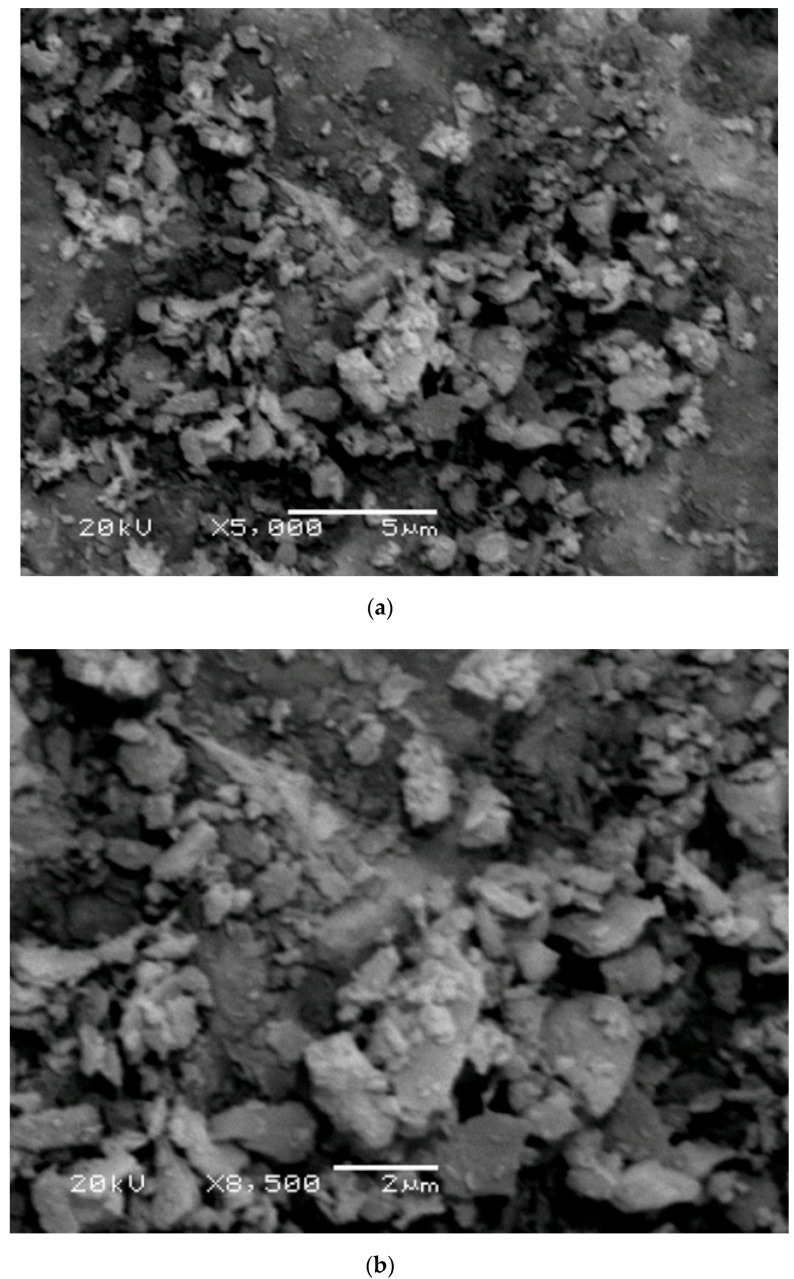

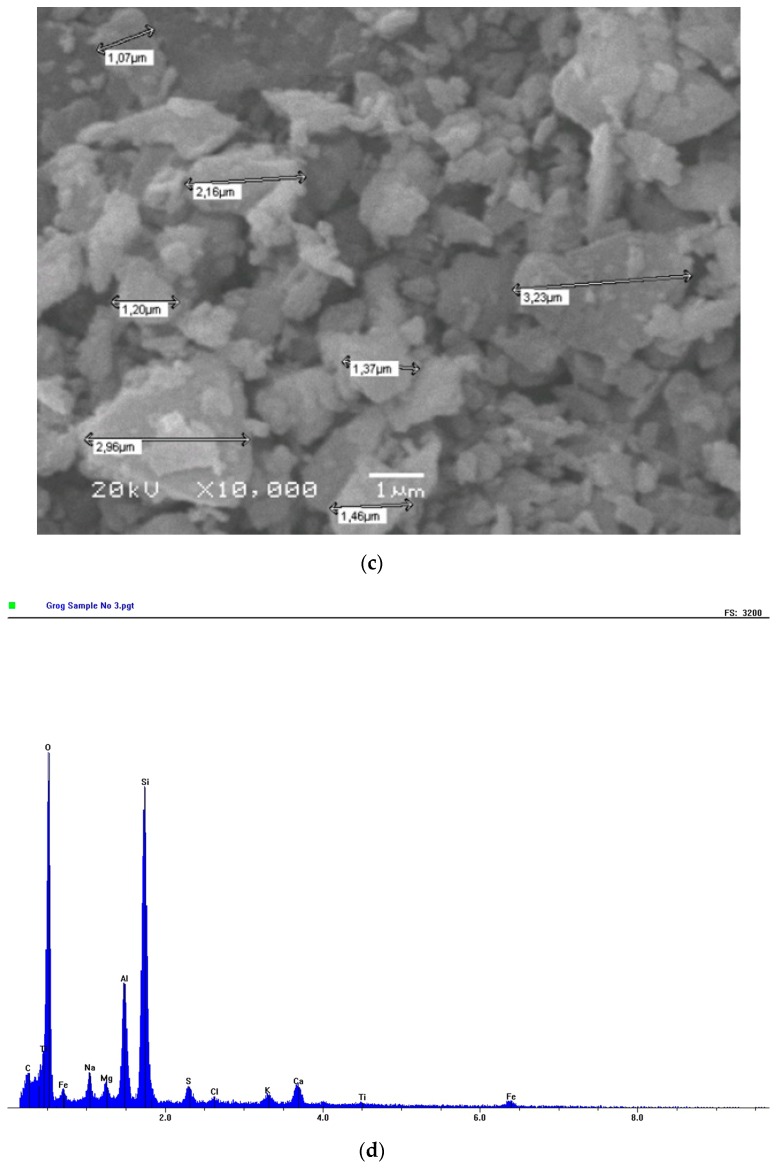
SEM images of the starting material (Grog) at magnification (**a**) ×5000, (**b**) ×8500, (**c**) ×10,000, and (**d**) its EDX analysis.

Figure 4. illustrates the microstructure morphology of Grog as well as their microanalysis (EDX). SEM images show the irregularly and angularly shaped microstructure morphology with a flaky grains appearance and an approximately 2.5 μm particle size distribution. In addition, the EDX shows the presence of silicon (Si), aluminum (Al), oxygen (O), and iron (Fe) as the main constituents of starting material with a minor presence of sodium (Na), calcium (Ca), and magnesium (Mg). The results obtained from SEM observation are consistent with the oxides composition analysis (XRF) given in Table 1 and the particle size distribution range, as shown in Figure 1.

On the other hand, the concentrations of alumina and silica in the form of Al^3+^ and Si^4+^ ions which leached into the solution and were used to measure the reactivity of Grog for the geopolymerization reaction were 30 and 82.60 mg/L, respectively. As expected, the concentration of Al^3+^ ions was much lower than that of Si^4+^ ions. This phenomenon is mainly due to a lower alumina content in the Grog, at approximately one-third of the silica content. Furthermore, the weight percentage of Si^4+^ ions leached into solution was about 16.52% of the total silica content in the Grog. This was mainly due to the considerable percentage of crystalline silica in the quartz phase, which reflects the total number of dissolved Si^4+^ ions after the geopolymerization reaction. In particular, the lower amorphous fraction of silica ions relied upon the mix design of the clay bricks raw meal and the sand that was used as a non-plastic filler in an additional step during the clay brick manufacturing process. In general, the extent of dissolution of Al^3+^ and Si^4+^ from source materials at a lower concentration of alkaline media at 1 M can be used to predict their behavior during a geopolymerization reaction in a high concentration (2.5–12 M) of alkaline media. In this way, the extent to which the starting material is affected by the alkaline solution can be evaluated [18]. Therefore, the alkali concentration of the geopolymer system in terms of the silica modulus molar ratio (Ms = SiO_2_/Na_2_O), H_2_O/Na_2_O, and liquid/solid ratio are significant parameters in controlling the process of dissolution of alumina and silica from the starting material (Grog), the subsequent geopolymerization rate, and the mechanical properties of the final hardened geopolymer product [31]. The results of the reactivity test indicated that the dissolution of the starting material (Grog) is sufficient in high alkaline concentration media rather than in low concentration media.

### 3.2. The Influence of Silica Modulus (Ms = SiO_2_/Na_2_O), H_2_O/Na_2_O Molar Ratio and L/S Ratio on Geopolymerization Rate

**Figure 5:**
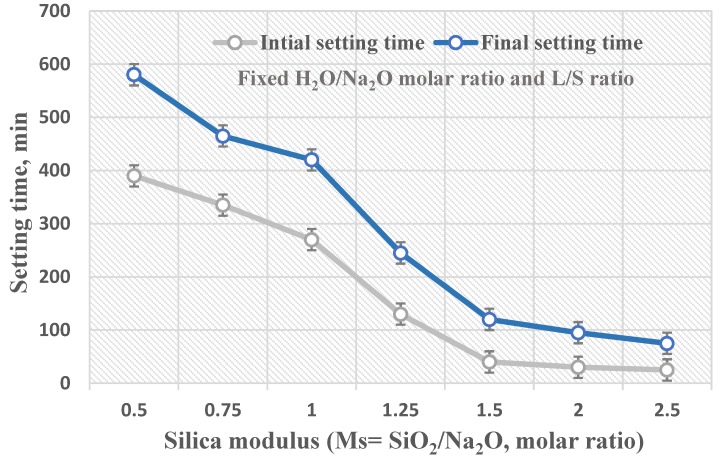
The geopolymerization rate in correlation with silica modulus (Ms = SiO_2_/Na_2_O, molar ratio).

The initial and final setting times of prepared geopolymer paste specimens generated by different alkaline activator solutions with a variation in the silica modulus (Ms = SiO_2_/Na_2_O) molar ratio ranging from 0.5–2.5 are presented in Figure 5. As can be seen, the geopolymerization rate and setting times are strongly dependent on the silica modulus molar ratio. In general, the setting times of the geopolymer pastes significantly decreased with an increase in the solution silica modulus molar ratio. The alkaline activator solution with a silica modulus molar ratio of Ms = 0.5 had the highest setting time value of around 400 min for the initial setting and about 600 min for the final setting time, which indicated a lower rate for the geopolymerization process. In contrast, the alkaline activator solution with a silica modulus molar ratio Ms = 2.5 had the smallest value of setting time at approximately 25 min for initial setting and 75 min for final setting, which indicated a faster rate for the geopolymerization process mainly due to the increases in free Si^4+^ ions in the geopolymer matrix. Furthermore, the alkaline activator solution became viscous as a result of an increasing silica modulus molar ratio, which was mainly related to increases in the content of sodium silicate in the alkaline activator. This effect decreases the workability of the paste and reduces the setting time. On the other hand, the alkalinity of the alkaline activator solution increased as the silica modulus molar ratio decreased, which is mainly caused by the presence of OH groups that play a key part in the dissolution of starting material and improve the solubility of silica and alumina during the geopolymerization process [11].

**Figure 6:**
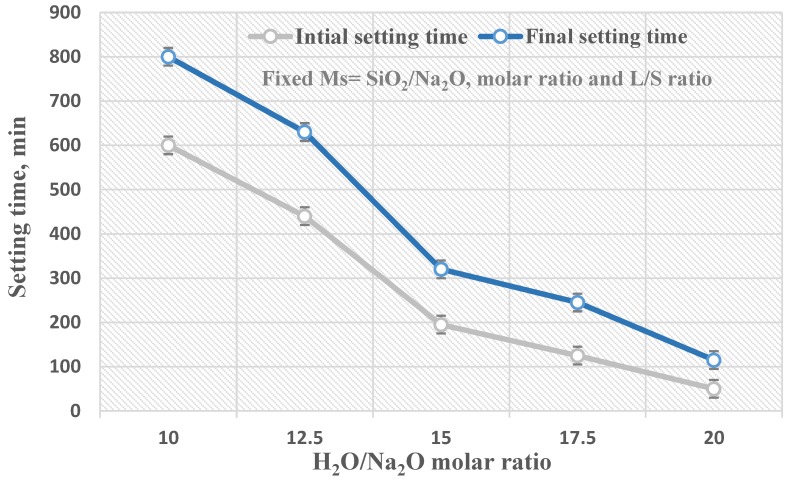
The geopolymerization rate in correlation with the H_2_O/Na_2_O molar ratio.

The setting times for geopolymer pastes prepared from different alkaline activator solutions with a fixed silica modulus molar ratio, Ms = 1.25, that had the highest physico–mechanical properties and a H_2_O/Na_2_O molar ratio ranging from 10–20 are shown in Figure 6. The setting time figure shows that the initial and final setting times decreased significantly as the H_2_O/Na_2_O molar ratio increased from 10 to 20. The initial setting times of the prepared geopolymer specimens with different H_2_O/Na_2_O molar ratios (10, 12.5, 15, 17.5, and 20) were 600, 440, 195, 125, and 50 min, respectively, and their final setting times were 800, 630, 320, 245, and 115 min, respectively. Consequently, as the molar ratio of H_2_O/Na_2_O decreased from 20 to 10, the viscosity of the alkaline solution increased, which led to a decrease of the workability of the geopolymer paste and a decrease of the ion mobility in the matrix, which was mainly connected with the increase of the setting time [11].

**Figure 7:**
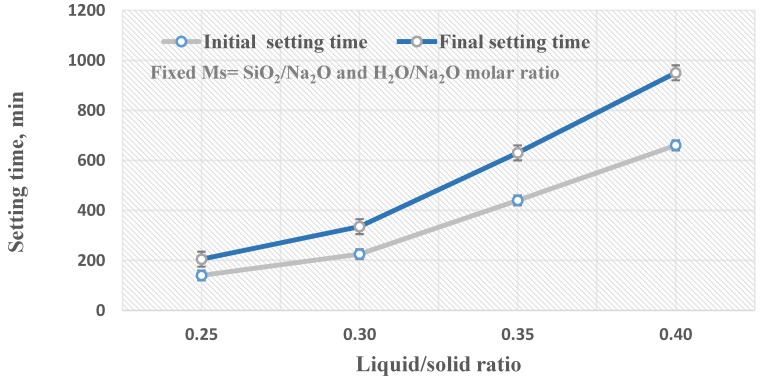
The geopolymerization rate in correlation with the liquid/solid ratio.

Figure 7 shows the rate of geopolymerization, in terms of the initial and final setting times, of geopolymer pastes made with different liquid/solid ratios using alkaline activator solutions with a fixed silica modulus molar ratio (Ms = 1.25) and a fixed H_2_O/Na_2_O molar ratio (12.5) which provided good physico–mechanical properties. As seen in Figure 7, the initial and final setting time increased remarkably as the liquid/solid ratio increased from 0.25 to 0.40 which indicates that the geopolymerization rate is also strongly affected by the liquid/solid ratio. This agrees with prior work that shows that as the liquid to solid ratio increases, the setting times of geopolymer paste also significantly increase [32]. Increasing the liquid/solid ratio of the geopolymer paste above 0.30 leads to a reduction of the alkali concentration of the geopolymeric system, which makes the paste more fluid and negatively affects the rate of geopolymerization reaction, which in turn leads to an increased setting time and reduced compressive strength for the final geopolymer product [33].

### 3.3. The Influence of Silica Modulus (Ms = SiO_2_/Na_2_O), H_2_O/Na_2_O Molar Ratio and L/S Ratio on the Physico–mechanical Properties of Geopolymers

**Figure 8:**
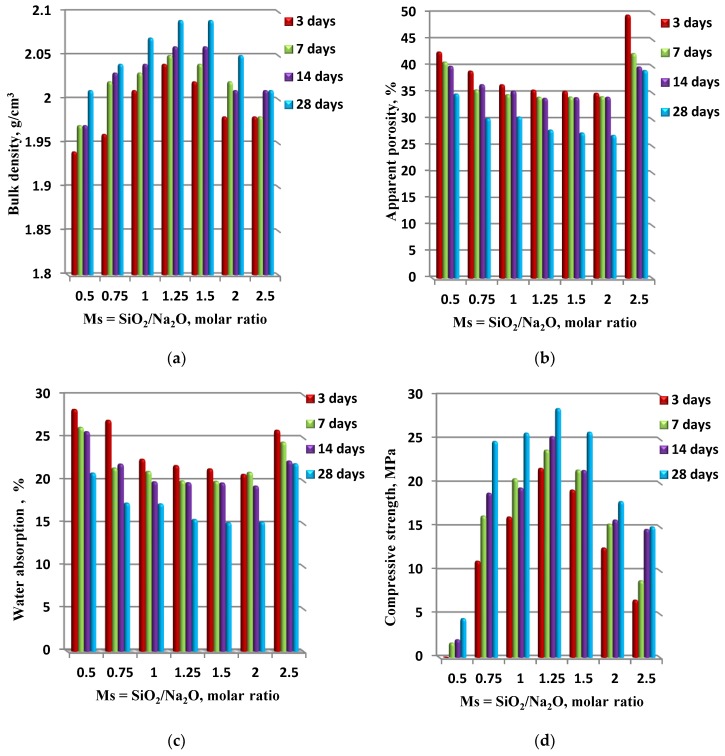
The physico–mechanical properties ((**a**) bulk density, (**b**) apparent porosity, (**c**) water absorption and (**d**) compressive strength) of synthesized geopolymer specimens in correlation with silica modulus (Ms = SiO_2_/Na_2_O, the molar ratio).

The physical and mechanical properties of grog-based geopolymers generated from different alkaline solution compositions, in term of various silica modulus (Ms = SiO_2_/Na_2_O) molar ratio ranging from 0.50–2.5, at different interval curing times are presented in Figure 8. As the results indicate, the bulk density of the geopolymer increased from 1.94 to 2.09 g/cm^3^ as the silica modulus molar ratio of the used alkaline solution increased from 0.5 to 1.25–1.5 and the curing time increased to 28 days for all synthesized geopolymer specimens. Consequently, as a result of the increasing bulk density of the geopolymer, the apparent porosity decreased from 42.7% to 27–28%. Moreover, the water absorption of the prepared geopolymer specimen decreased from 28.5% to 15% as the silica modulus of the alkaline solution increased from 0.50 to 1.25–1.5, and the curing time decreased as well. Consequently, as the physical properties of the geopolymer specimens were enhanced by increasing the silica modulus up to 1.5 and the curing time up to 28 days, the compressive strength improved. Hence, the compressive strength shows a corresponding increase up to a silica modulus of 1.25. After that, a reduction in the compressive strength can be observed. Furthermore, the compressive strengths after 28 days curing for specimens synthesized with alkaline activator solutions with a silica modulus of 1.25 and 1.5 were 28.5 and 25.8 MPa, respectively, and the strength of the geopolymer specimen made with a 0.5 silica modulus was much lower, at around 4.5 MPa. As seen in Figure 8, the physico–mechanical properties of synthesized geopolymer matrixes improved with the increasing silica modulus ranging from 0.75–1.5 and the optimum value at 1.25. It was previously reported that the increase of silica modulus usually leads to an improvement in the geopolymerization reaction rate, but it can reduce the geopolymerization rate at a high silicate concentration ratio of over 2.0. Subsequently, the results were in good agreement and had consistency with the previous investigation [34].

**Figure 9:**
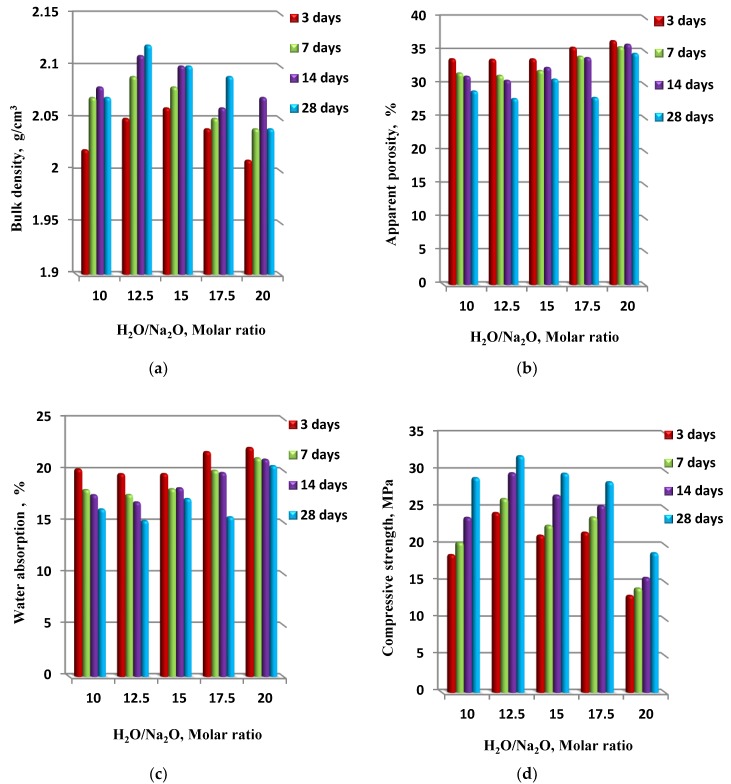
The physico–mechanical properties ((**a**) bulk density, (**b**) apparent porosity, (**c**) water absorption and (**d**) compressive strength) of synthesized geopolymer specimens in correlation with H_2_O/Na_2_O, the molar ratio.

The physico–mechanical properties of waste fired clay bricks-based geopolymers made using different alkaline solution compositions, in terms of various H_2_O/Na_2_O molar ratios of 10–20 and a fixed silica modulus Ms = 1.25 at different interval curing times, are presented in Figure 9. There were clear differences in the physico–mechanical properties, which shifted with an increasing H_2_O/Na_2_O molar ratio of up to 20. From the presented data, the highest bulk density value was 2.12 g/cm^3^, the lowest apparent porosity percentage was 27.8%, and the water absorption was 15.1% for the geopolymer specimen synthesized using a 12.5 H_2_O/Na_2_O molar ratio and with 28 days of curing time. Accordingly, the highest compressive strength 32 MPa was measured for the same specimen after 28 days of curing time. It is also notable that the physico–mechanical properties were enhanced in a range of H_2_O/Na_2_O molar ratio of 10–12.5. On the other hand, the high-water content at the molar ratio H_2_O/Na_2_O of 20 resulted in the lowest compressive strength, at around 19 MPa.

**Figure 10:**
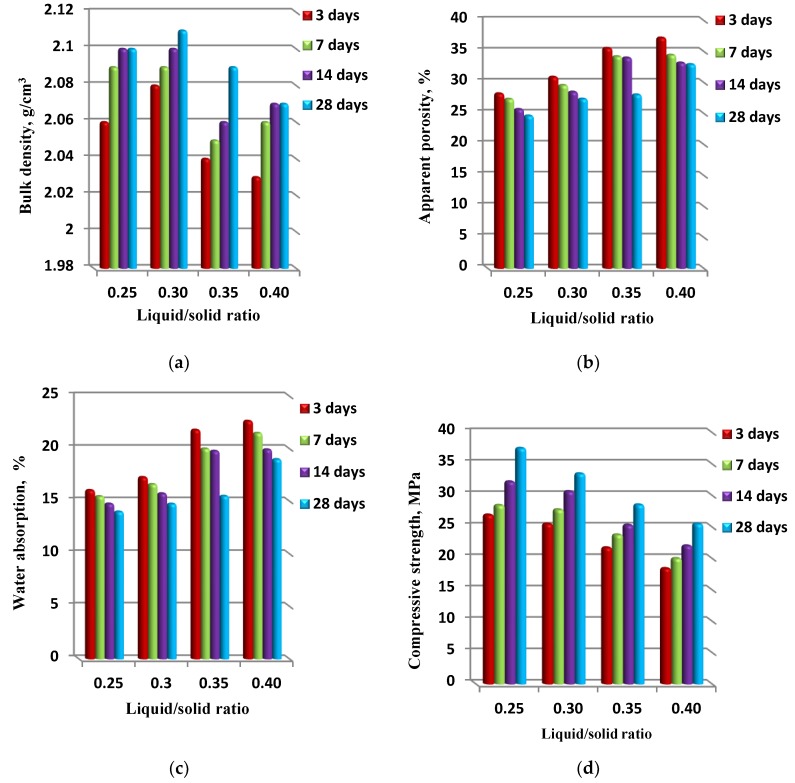
The physico–mechanical properties ((**a**) bulk density, (**b**) apparent porosity, (**c**) water absorption and (**d**) compressive strength) of synthesized geopolymer specimens in correlation with a liquid/solid ratio.

The physico–mechanical properties of waste fired clay bricks-based geopolymers produced using different liquid/solid ratios ranging from 0.25–0.40, a 1.25 silica modulus, a 12.5 H_2_O/Na_2_O molar ratio, and different interval curing times are presented in Figure 10. It is well known that the liquid/solid ratio of any binder material system is one of the most important factors, along with the curing conditions, that significantly influences the physico–mechanical properties of the resulting hardened paste [11]. As the results indicate, the physico–mechanical properties of the geopolymer specimens in terms of the bulk density, apparent porosity, water absorption, and compressive strength were enhanced when the liquid/solid ratio range was 0.25–0.30. As expected, above the liquid/solid ratio of 0.30, there was a reduction in the physico–mechanical properties of the geopolymer specimens. Usually, with an increase in the liquid/solid ratio, there is an improvement in the workability of a geopolymer binder system’s paste, mortar, or concrete. However, it should be noted that in the most cases, the increase in liquid/solid ratio consequently leads to an increase in the total porosity of the system, which reflects negatively on the compressive strength behavior of the hardened paste. A significant difference can be observed between the highest 28 days cured compressive strength value recorded at around 37.5 MPa for the geopolymer specimen produced by a 0.25 liquid/solid ratio, and the lowest 28 days cured compressive strength value, recorded at around 25.5 for the geopolymer specimen made with a 0.40 liquid/solid ratio. When high liquid/solid ratios in the 0.35–0.40 range were applied, there was an increase in the amount of fluid medium rather than solid content in the paste, as a result of contact between the alkaline activator solution and the reacting solid material, which was limited due to the large volume of fluid medium. Furthermore, the dissolution of solid material was slow, and the geopolymerization rate was slow. This explains the low 28-days compressive strength of a geopolymer specimen with a liquid/solid ratio of 0.40. In contrast, when lower liquid/solid ratios in the 0.25–030 range were used, the solid material content increased in the geopolymer paste. The interaction between the alkaline activator solution and reacting solid material led to an enhancement in the physico–mechanical properties of the geopolymer specimens. Therefore, an optimal liquid/solid ratio in the 0.25–0.30 range exists for waste fired clay bricks based geopolymers [11].

### 3.4. FTIR of the Generated Geopolymers

**Figure 11:**
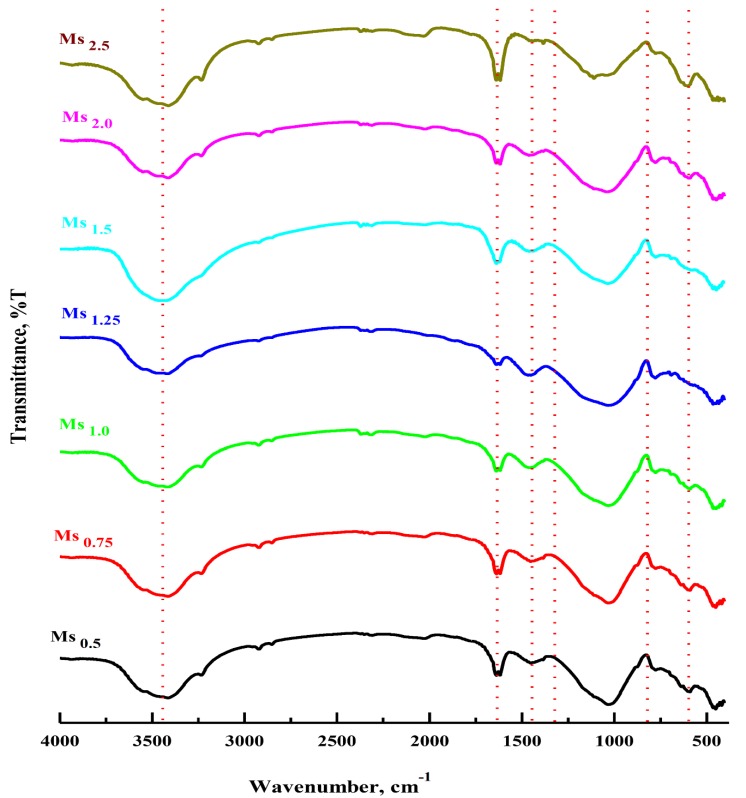
FTIR spectra of synthesized geopolymer specimens in correlation with the silica modulus (Ms = SiO_2_/Na_2_O, the molar ratio) of Ms_0.5_, Ms_0.75_, Ms_1_, Ms_1.25_, Ms_1.5_, Ms_2.0_ and Ms_2.5_, respectively.

Figure 11 presents the FTIR spectra of the generated grog based geopolymers made with various alkaline solutions, in term of silica modulus (Ms = SiO_2_/Na_2_O) molar ratios, at a curing time of 28 days. In the FTIR spectra of the geopolymer products, the band centered at about 460 cm^−1^, corresponding to Si–O bending. Other bands, attributed to the formation of the geopolymer structure are at about 3450 and 1635 cm^−1^ and are associated with the bending and stretching bands of H_2_O molecules that have been either surface absorbed in the geopolymer structure cavities or bonded physically to the silanol surface groups (≡Si–OH….H_2_O and ≡ Al–OH…..H_2_O) [35,36]. Furthermore, weak adsorption bands at 600 and 780 cm^−1^ are assigned to Si–O–Al bending, indicating the formation of the main geopolymer network structure that was generated during the geopolymerization reaction of a silica aluminate material in a highly alkaline solution. The main band centered at 1100 cm^−1^ in the grog spectrum shifted to a lower frequency at ≈ 850 cm^−1^ and broadened to ≈ 1350 cm^−1^, confirming the formation of a new geopolymer product. Furthermore, the FTIR spectra of the prepared geopolymers showed a distinct intense broad band at approximately 850 and 1350 cm^−1^ for all samples associated with the asymmetric vibration of Si–O–T units of the silicate framework of the formed geopolymer network. Moreover, this peak is often used to determine the degree of polymerization because it is easier to interpret than the Si–O–Si bonding peak at 450 cm^−1^ [37]. Moreover, the observed shift towards a higher wavenumber can be connected with the generation of more condensed Si–O–Si units in the geopolymer network. Based on the data presented in the FTIR spectra, broadening in this band increases as a result of increasing the silica modulus of the used alkaline solution and the broadest band occurs in the geopolymer sample prepared with an alkaline solution with a 1.25 silica modulus molar ratio [38]. The FTIR bands at approximately 1450 cm^−1^ assigned to the vibration mode of O–C–O bonds, and two minor peaks at 2355 cm^−1^ assigned to the stretching vibration mode of C=O bonds are related to the carbonate formation process due to the reaction of alkali metal hydroxide with atmospheric CO_2_ to produce Na_2_CO_3_, which was observed in all spectra of the generated geopolymer samples [10]. On the other hand, the intensity of these carbonate bands was reduced as the silica modulus increased and it disappeared at higher silica modulus values (Ms ≥ 1.5), indicating less carbonate formation in the geopolymer matrix.

**Figure 12:**
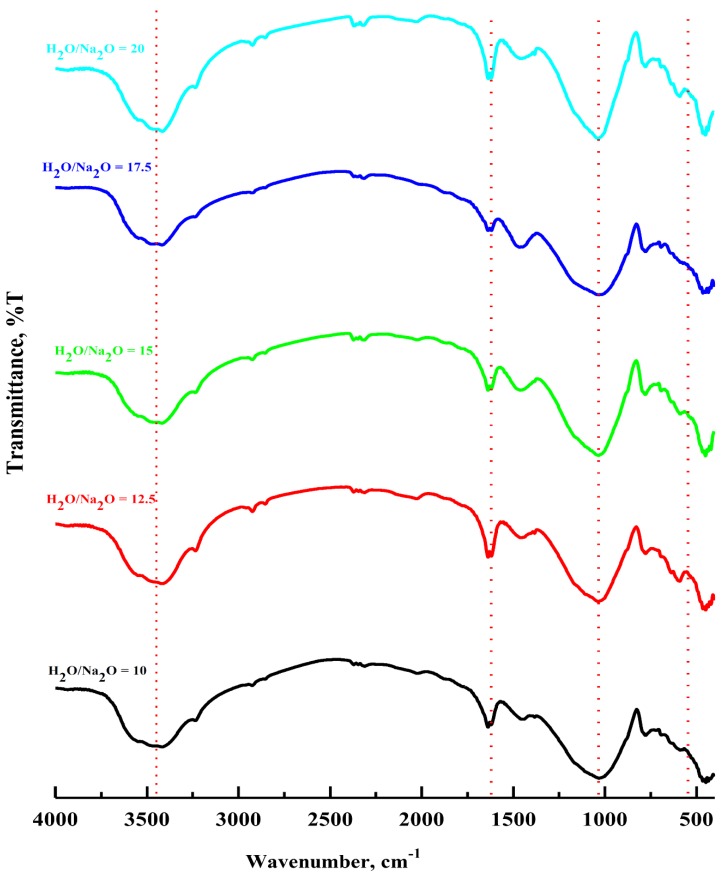
FTIR spectra of synthesized geopolymer specimens in correlation with H_2_O/Na_2_O, the molar ratio, of 10; 12.5; 15; 17.5 and 20, respectively.

Figure 12 shows the FTIR spectra of the prepared grog based geopolymers using various alkaline solutions, in terms of H_2_O/Na_2_O ratio molar ratios, at 28 days curing time. The broad band appearing at 850–1350 cm^−1^, attributed to the asymmetric stretching vibration of Si–O–Si, indicates the formation of a geopolymer network structure, which decreases as the molar ratio of H_2_O/Na_2_O increases from 10 to 20. Moreover, the bending vibration of Si–O–Si was identified at 460 cm^−1^ and the peak at approximately 650 cm^−1^ which corresponds to the Si–O–Al bending vibration, were also observed, especially at a 12.5 H_2_O/Na_2_O molar ratio. The geopolymerization degree affected the stretching vibration band of Si–O–Si and the wavenumbers of the absorption bands increased with the degree of geopolymerization. Furthermore, the intensity of the stretching vibration of O–C–O detected at 1420 cm^−1^ remarkably increased as the molar ratio of H_2_O/Na_2_O increased from 10 to 20, which was mainly related to an increase in the ion mobility of the geopolymer samples and positively reflected on the rate of the carbonation process. In addition, the broad bands which appeared at 3450 cm^−1^ and 1650 cm^−1^, mainly assigned to entrapped water molecules in the geopolymeric network, increased as a result of increasing the amount of the water molecules in the geopolymerization reaction through increasing the molar ratio of H_2_O/Na_2_O.

### 3.5. XRD of the Synthesized Geopolymers

**Figure 13:**
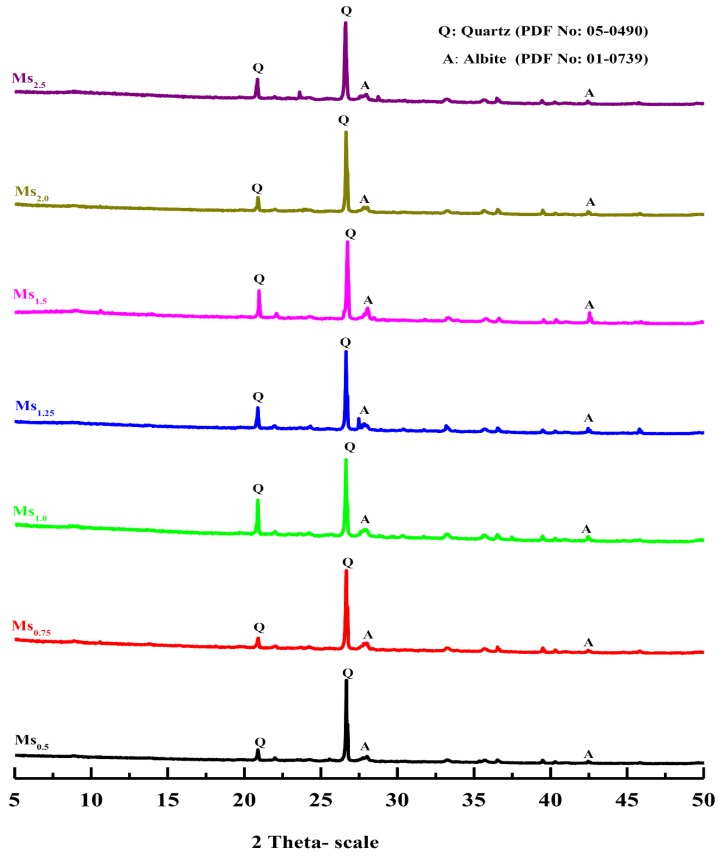
X-ray diffraction pattern of geopolymers prepared with different silica modulus (Ms = SiO_2_/Na_2_O, the molar ratio) of Ms_0.5_, Ms_0.75_, Ms_1_, Ms_1.25_, Ms_1.5_, Ms_2.0_ and Ms_2.5_, respectively.

**Figure 14:**
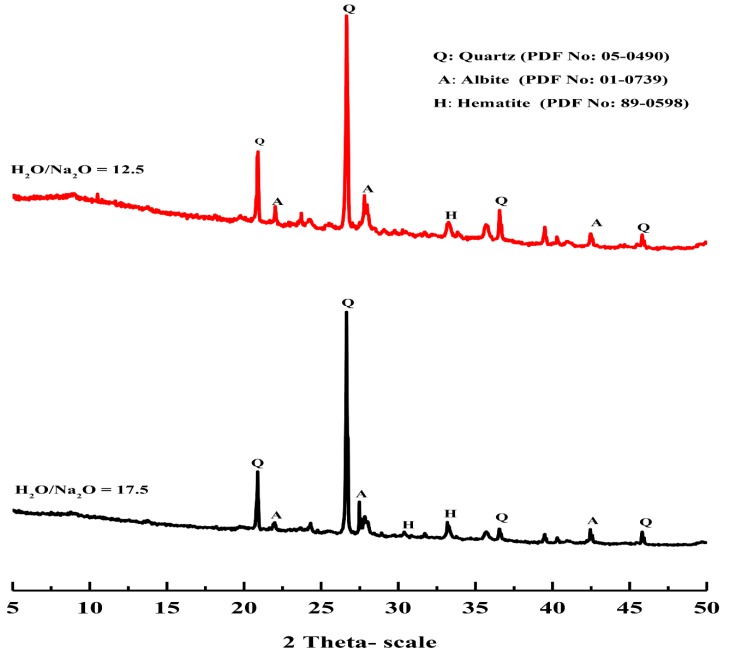
X-ray diffraction pattern of geopolymers prepared with different H_2_O/Na_2_O molar ratios of 12.5 and 17.5, respectively.

Figure 13 shows the XRD diffractograms of different geopolymer specimens synthesized with various alkaline solutions in terms of various silica modulus (Ms = SiO_2_/Na_2_O) molar ratios, with 28 days of curing time. The XRD pattern shows that the main crystalline phases detected in all geopolymers were quartz (SiO_2_) and albite (NaAlSi_3_O_8_). The crystalline phase observed in the XRD pattern of the geopolymer specimens mainly contributed to the partial dissolution of the total SiO_2_ content of the starting material (Grog) that accumulated from two different sources (clay and sand) through a raw meal mix preparation step in the clay brick manufacture process. Although the conventional geopolymer mainly consists of amorphous phases [30], some crystalline phases might be identified in the XRD pattern of geopolymer generated from some starting materials such as circulating fluidized bed combustion (CFBC) bottom ashes [39], and volcanic ash [40,41]. Otherwise, the disappearance of crystalline iron oxide peaks upon alkaline activation of the starting material was mainly related to the dissolution of iron oxide into ionic monomers or oligomers, which then polymerized based on the pH [42]. Nevertheless, many recent studies had reported that iron-rich materials such as fly ashes can be activated in alkaline media for engineering applications [43,44]. Furthermore, the XRD pattern also indicated that the intensity of crystalline phases corresponding to quartz and albite in the geopolymer specimens decreased as the silica modulus of used alkaline solution decreased, and the alkalinity of the solution increased as a result. Therefore, the relatively low and mild alkalinity of the alkaline solution hardly dissolved the crystalline phases in the waste fired clay bricks material. Moreover, the resultant interface structure between the produced geopolymer aluminosilicate network gel and remaining unreacted crystalline particles in the geopolymer matrix was expected to have a positive influence in the binding capacity of the matrix and have a significant bearing on the overall physico–mechanical properties of the resulting geopolymer specimen [45,46]. On another hand, the XRD pattern represented in Figure 14 of the geopolymer samples prepared using different H_2_O/Na_2_O molar ratios also shows the presence of some diffraction peaks attributed to the crystalline phases that remained as unreacted residues from the starting material. As mentioned before, the effect of the H_2_O/Na_2_O molar ratio on the geopolymer system can be attributed to the mobility of the ions in the synthesized matrix, which can be significantly observed through the physico–mechanical properties.

### 3.6. SEM of the Synthesized Geopolymers

**Figure 15:**
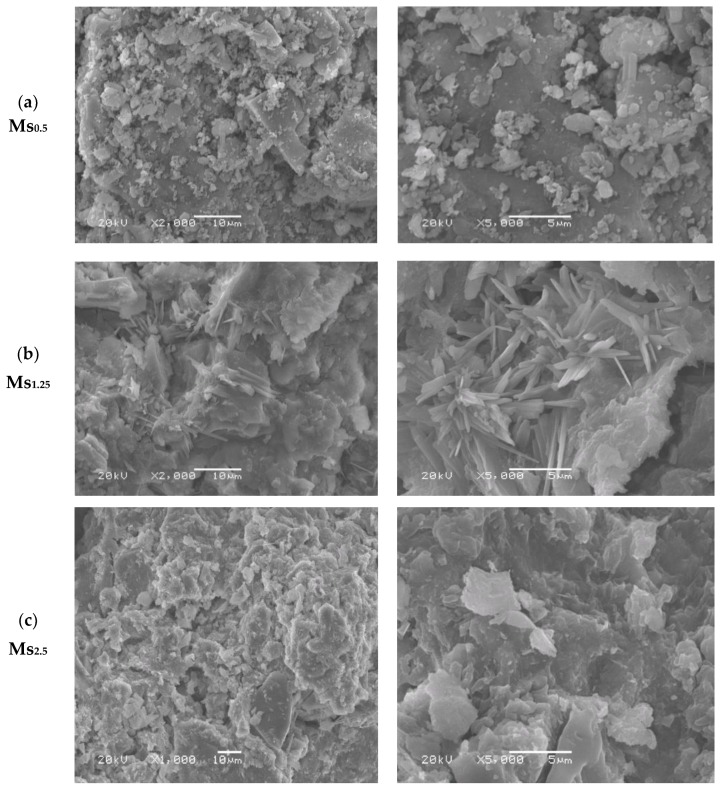
SEM micrograph of geopolymers prepared with a different silica modulus Ms = SiO_2_/Na_2_O molar ratio ((**a**) 0.5, (**b**) 1.25 and (**c**) 2.5) at different magnification.

**Figure 16:**
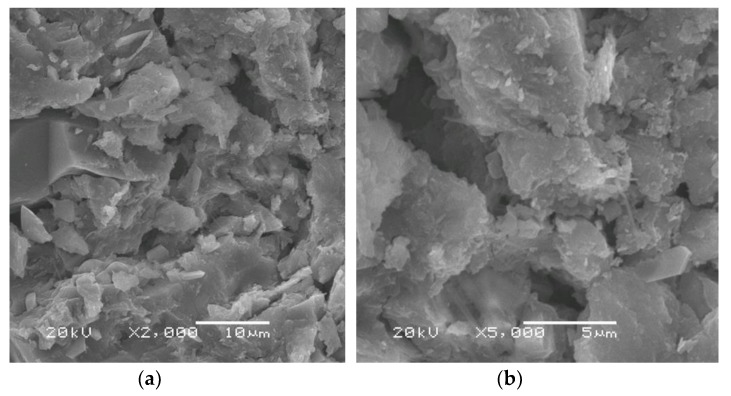

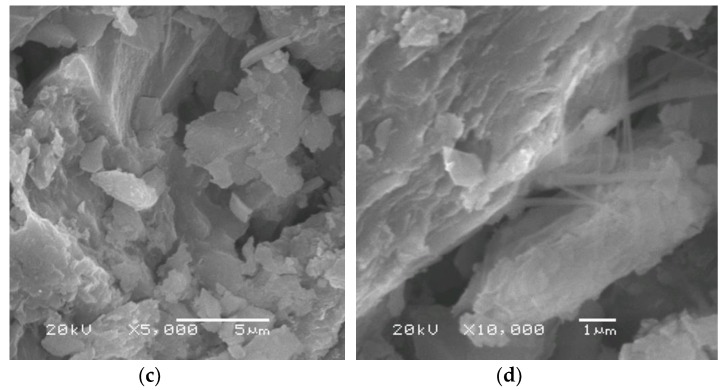
SEM images of geopolymers prepared with silica modules Ms = 1.25, a H_2_O/Na_2_O molar ratio = 12.5, and an L/S ratio = 0.30. ((**a**) × 2000, (**b**) × 5000, (**c**) × 5000 and (**d**) × 10,000).

Figure 15 shows the SEM micrographs of the geopolymer samples generated with various silica modulus solutions (Ms = 0.5, 1.25 and 2.5) and cured for 28 days at ambient temperature (20–25 °C). The geopolymer sample prepared with Ms = 0.5 shows a microstructure quite similar to SEM of the starting raw material, with a few characteristics of amorphous phase microstructure related to a geopolymer matrix. Moreover, some unreacted grog particles are clearly visible in the SEM image, which illustrates the insufficiency in the geopolymerization reaction at this level of the silica modulus molar ratio. Furthermore, this geopolymer sample had the lowest recorded value of compressive strength (4.5 MPa) after 28 days of curing and a showed much less compacted microstructure composed of a large number of particles barely consolidated with each other, creating a very weak compacted microstructure [10]. On the other hand, the geopolymer sample generated using an alkaline solution with Ms = 1.25 showed an irregularly fibrous shaped structure which mainly corresponds to a geopolymer amorphous network microstructure. It was formed during production of a geopolymer gel which filled and was deposited in pore spaces between the particles to form a denser microstructure and improve the overall physico–mechanical properties. Furthermore, the interaction between the produced geopolymer gel and the unreacted residues from raw material had a significant influence on the microstructure and on the overall development of compressive strength [15]. As a result, this geopolymer specimen had the highest recorded value of compressive strength (28.5 MPa) after 28 days of curing time and showed a highly compacted microstructure in comparison with the other geopolymer specimens. In addition, SEM images show the presence of some rod shapes in the microstructure which related to efflorescence formation due to the carbonation process of unreacted sodium oxide. This efflorescence was also confirmed by FTIR spectrums. On the other hand, the SEM micrographs of geopolymer specimen prepared using alkaline solution with Ms = 2.5 showed an irregular microstructure and morphology, which confirms the presence of a significant amount of remaining unreacted raw material particles as a result of the insufficient geopolymerization reaction. These microstructural differences observed in SEM micrographs were significant enough to justify the difference in physico–mechanical behavior of geopolymer specimens prepared using various silica modulus molar ratio alkaline solutions. Figure 16 shows the SEM micrographs of the geopolymer samples prepared with Ms = 1.25, a 12.5 H_2_O/Na_2_O molar ratio, and a 0.3 liquid/solid ratio as well as being cured for 28 days at an ambient temperature (20–25 °C). Based on the SEM observation of this geopolymer sample, a more amorphous aluminosilicate gel phase with a smaller amount of unreacted particles could be observed, which indicated a significantly higher geopolymeric microstructure. Moreover, the sample homogeneity and a less porous microstructure were the predominant features of this SEM micrograph in comparison with the geopolymer specimens manufactured under different conditions in this study.

### 3.7. Thermal Analysis of the Geopolymer Synthesized at Optimum Condition

**Figure 17:**
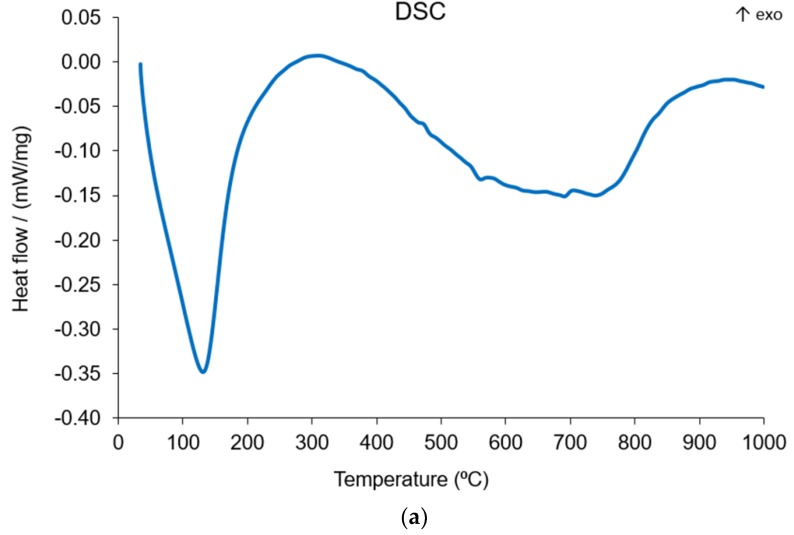

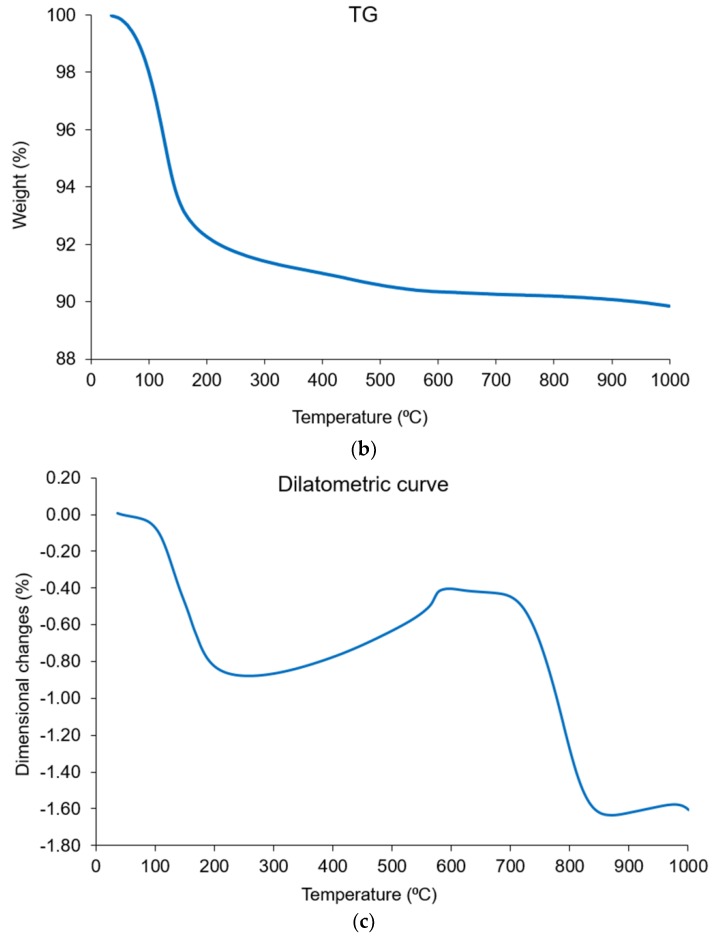
(**a**) DSC, (**b**) TG, and (**c**) Dilatometric curves of the optimum geopolymer specimen.

The DSC, TG, and dilatometric curves of the optimum geopolymer specimen (SiO_2_/Na_2_O = 1.25, H_2_O/Na_2_O = 12.5, liquid/solid ratio = 0.30) are presented in Figure 17. DSC analysis of geopolymer behavior from room temperature to 300 °C showed a large endothermic peak. In this temperature range, on the TG curve, an intensive weight loss was recorded. Additionally, the dimensional changes presented about 1% linear shrinkage. This observed effect is well known and is associated with the removal of absorbed water and water contained within the pore network [47,48,49]. Moreover, the sample size expansion recorded on the dilatometric curve (Figure 17) in the temperature ranged from 300 °C to 600 °C can be attributed to a dehydroxylation process and the polymorphic α-β transition of an unreacted quartz phase or glass transition in the geopolymer matrix [50].

Furthermore, this effect is associated with a low mass decrease and a second endothermic reaction. There was also a rapid shrinkage between 700 and 870 °C, which was characteristic of structural reorganization. This result and the lack of changes in the TG curve confirm these phenomena.

## 4. Conclusions

The current study investigates the optimum geopolymerization reaction conditions for a safe recycling process of the starting raw material (Grog). At this point, several geopolymerization factors affecting the final properties of geopolymer specimens were investigated and examined. The properties of the synthesized Grog-based geopolymers were mainly influenced by the silica modulus (Ms), H_2_O/Na_2_O molar ratio of the alkaline solution, and the liquid /solid ratio. The recommended optimum geopolymer conditions were selected based on the achieved overall properties of the synthesized geopolymer specimens. As the results indicate, the following conclusions were reached:

- The optimum silica modulus and H_2_O/Na_2_O molar ratio of the alkaline solution were Ms = 1.25 and 12.5, respectively.
- The optimum liquid/solid ratio was in the range 0.25–0.30.
- The 28 days compressive strength of the Grog-based geopolymer sample generated under optimum conditions was 37.5 MPa in comparison with 5 MPa, which generated under random conditions.

This study proposes a safe recycling process for waste material (Grog), which otherwise causes a lot of environmental problems, through an optimum condition geopolymerization process. Consequently, the generated binder material provides an alternative binder material that can be used in many applications in the construction sector.

## Figures and Tables

**Table 1:** Chemical oxide composition of waste fired clay bricks (Grog), mass %.

Oxides	SiO_2_	Al_2_O_3_	Fe_2_O_3_	CaO	Na_2_O	K_2_O	TiO_2_	MnO	MgO	SO_3_	BaO	P_2_O_5_	Cl	LOI ^a^
Grog	50.16	15.95	15.09	4.39	2.43	1.48	2.10	0.16	2.13	2.78	0.06	0.24	0.46	2.15

^a^ loss on ignition at 950 °C.
